# 

**Published:** 1873

**Authors:** 


### TABLE OF CONTENTS.

### ORIGINAL COMMUNICATIONS.

The Origin, Nature and Treatment of Fever, etc. By Ch. Rauschenberg, M.D........303

Eclampsia—Case, Cause, Treatment. By K. P. Moore, M,D........317

Nature and Art. By Robert Battey, M D........323

The Therapeutic Effects and Uses of Mercury, etc. By Wm. H. Doughty, M.D........325

### SELECTIONS.

Questions Medico-Legal........340

Twenty-first Annual Meeting of the American Pharmaceutical Association........351

An Address on Obstetric Medicine........363

Ovariotomy by Enucleation........376

Foreign Correspondence........381

Simulated Amaurosis........386

Physiological Effects of Double Ovariotomy........394

What is Cincho-Quinine?........395

International Medical Congress at Vienna........398

The Profession of Nursing........400

The Laws of Epidemics and Preventives........401

Administration of Perchloride of Iron........402

### EDITORIAL AND MISCELLANEOUS.

To the Patrons of the Journal........403
City Hospital........404
Choked on Potato and Beef........406
A Case of Triplets—Saccharated Calomel........407
Bibliographical........408
Call for a Meeting of Surgeons of the Confederate Army........413

☞ *The Editors are not responsible for the views of Contributors.*

